# WISp39 and Hsp90: actin' together in cell migration

**DOI:** 10.18632/oncotarget.4934

**Published:** 2015-07-21

**Authors:** Rati Fotedar, Robert L. Margolis

**Affiliations:** Sanford-Burnham-Prebys Medical Discovery Institute, La Jolla, CA, USA

**Keywords:** Chromosome Section, WISp39, motility, Coronin 1B, Cofilin, Hsp90

Cell motility is an actin dependent process requiring the formation and extension of lamellipodia or filopodia, and is absolutely essential for many cellular processes, especially for morphogenesis during development. During lamellipodial extension, actin dynamics involve switching between branch formation at the leading edge and proximal severing of existing actin filaments [1]. Actin branch formation by the Arp2/3 complex changes cell shape and produces protrusions. In addition, the actin depolymerizing factor Cofilin maintains actin turnover by severing and depolymerizing actin filaments [2]. However, a molecular understanding of how actin treadmilling, driven by Arp2/3 dependent branching at the leading edge, is balanced with Cofilin-dependent severing toward the cell interior has remained incomplete.

What is known is that Arp2/3 dependent actin assembly and Cofilin dependent disassembly are coordinately regulated at the leading edge by Coronin 1B, which binds the Arp2/3 complex in a phosphorylation-dependent manner. When dephosphorylated, Coronin 1B inhibits the binding of the Arp2/3 complex to actin, but phosphorylation by protein kinase C at Ser2 reduces its association with the Arp2/3 complex, enabling actin branch formation [3]. Thus, the regulation of phosphorylated Coronin 1B is essential to control Arp2/3 complex activity and ultimately the rate of actin nucleation and branching at the leading edge.

Cofilin is also regulated by phosphorylation but, in contrast to Coronin 1B, it is inactivated by phosphorylation, and becomes active through Slingshot phosphatase (SSH) dephosphorylation [2]. The first indication that Coronin 1B regulates Cofilin came from work showing that Coronin 1B interacts with and is dephosphorylated by SSH, and that this interaction then promotes the dephosphorylation and activation of Cofilin [3]. One crucial missing piece of the puzzle is how the Coronin 1B and SSH interaction is regulated to control dephosphorylation of Cofilin, thus sustaining leading edge actin dynamics.

Our recent study has revealed two new important players in cellular motility. We have found that WISp39 (Waf1 Cip1 stabilizing protein 39), an Hsp90 binding protein we had discovered [4], coordinates Coronin 1B, Arp2/3 complex and Cofilin activity in the lamellipodia to achieve directed cell motility [5]. WISp39 is recruited to actin-enriched cell protrusions which contain the Arp2/3 complex and Coronin 1B. Its knockdown results in the loss of directional motility and cell polarity, accompanied by profound changes in cell morphology [5]. Rescue of WISp39 function in WISp39 siRNA-treated cells restores directionality of motility to control levels. Most interestingly, we found that Hsp90 is an important component of the regulatory complex, as WISp39 mutants that do not bind Hsp90 cannot rescue the directionality defect [5]. Although Hsp90 has been shown to bind actin, and to increase the stability of proteins that control actin dynamics, its role in cell motility has not received much attention. Our study has thus not only revealed a novel mechanism by which Hsp90 participates in actin-mediated cell motility through WISp39, it has also revealed a central role for Hsp90 in this function.

**Figure 1:**
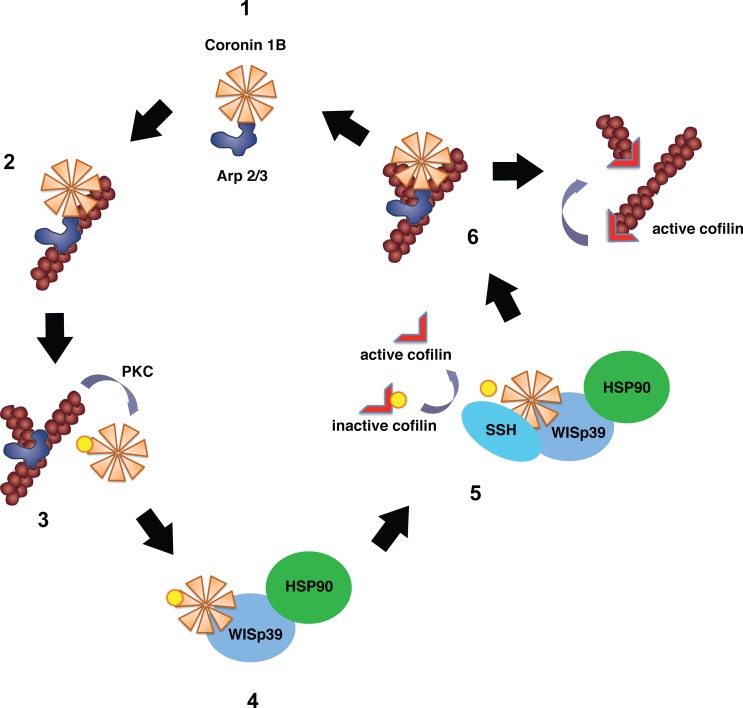
Model of WISp39 function Dephosphorylated Coronin 1B binds the Arp2/3 complex (step 1) and localizes it to the side of the actin filament (step 2) preventing branching by the Arp2/3 complex. Coronin 1B phosphorylated by PKC on Ser2 loses its affinity for the Arp2/3 complex, allowing Arp2/3 complex-mediated branching (step 3). Phosphorylated Coronin 1B binds WISp39 in a complex with Hsp90 (step 4, our publication) promoting its association with SSH. SSH then dephosphorylates Coronin 1B and Cofilin (step 5) [3]; our publication). Dephosphorylated Coronin 1B binds the Arp2/3 complex and removes it from the branch (step 6). The destabilized branch is then severed by active Cofilin. See original reference for more details.

Using biochemical approaches to dissect the interactions of WISp39, phospho-Coronin 1B, SSH and Hsp90, we have demonstrated that WISp39 may be the scaffold that orchestrates the activity of key regulators of actin dynamics (Arp2/3 complex, Coronin 1B and Cofilin) to maintain lamellipodia protrusion and directed motility as shown in Figure 1. Our data show that Coronin 1B exists in two distinct complexes; dephosphorylated Coronin 1B bound to the Arp2/3 complex without SSH or WISp39, and phosphorylated Coronin 1B bound to WISp39 and SSH but not the Arp2/3 complex. Specifically, loss of WISp39 reduces the association of phospho-Coronin 1B with SSH, increases Cofilin phosphorylation thereby decreasing Cofilin activation, and reduces the localization of the Arp2/3 complex at the leading edge, causing a loss of directed cell motility. The morphological defects exhibited by WISp39-depleted cells are rescued by coexpression of Coronin 1B and constitutively active Cofilin(S3A) [5]. Consistent with these results, WISp39 knockdown yields a phenotype similar to Coronin 1B knockdown [5], with the exception that WISp39 knockdown cells migrate faster than controls, whereas Coronin 1B knockdown cells migrate slower [3]. Importantly, Cofilin knockdown cells also migrate faster, similar to WISp39 knockdown cells [6]. Therefore, WISp39 knockdown presents a phenotype with attributes of both Coronin 1B knockdown and Cofilin knockdown [5].

We have previously demonstrated that WISp39 interacts with cyclin-dependent kinase inhibitor p21 and Hsp90 in a trimeric complex that stabilizes p21 against degradation [4]. That study highlighted a role for WISp39-Hsp90 complex in regulating both the basal levels of p21 and the increase in p21 protein in response to ionizing radiation [4, 7]. However, we found that knockdown of p21 with siRNA does not influence any parameters of motility, showing that WISp39-Hsp90 complex has two discrete functions, one involving the stabilization of p21, and the other involving interaction with Coronin 1B to regulate cell migration [5].

Our data support a key role for WISp39 in regulating actin dynamics to sustain directed cell motility. We suggest that WISp39 and its binding partner Hsp90 act as a crucial scaffold that integrates Coronin 1B, SSH and Arp2/3 complex at the leading edge.
